# Curious Case of Superfitial Spreading Cervical Squamocellular Carcinoma with Adnexal Involvement

**DOI:** 10.3390/medicina58111655

**Published:** 2022-11-16

**Authors:** Milan Dokić, Svetlana Milenković, Ljubiša Jovanović, Branislav Milošević, Luka Andrić, Dušan Šaponjski, Vesna Kesić

**Affiliations:** 1 Clinic for Gynecology and Obstetrics, University Clinical Center of Serbia, 11000 Belgrade, Serbia; 2 Medical Faculty, University of Belgrade, 11000 Belgrade, Serbia; 3 Department of Pathology and Medical Cytology, University Clinical Center of Serbia, 11000 Belgrade, Serbia; 4 Center for Radiology Imaging-Magnetic Resonance, University Clinical Center of Serbia, 11000 Belgrade, Serbia

**Keywords:** cervical cancer, superfitial spreading

## Abstract

*Background and Objectives*: Cervical squamous cell carcinoma (SCC) usually showed an infiltrative growth pattern into endocervical stroma. In rare cases, SCC spreads superficially as an intraepithelial lesion to proximal uterine segments, and more rarely, involves invasive and more aggressive behavior on secondary sites. *Materials and Methods*: In this study, we present the case of an interesting form of cervical SCC growth and we discuss the possible reasons for that presentation. *Results*: After clinical examination and repeated histomorphological analysis, we found remarkable cervical epithelial dysplasia (a high-grade squamous intraepithelial lesion—H-SIL). A histopathology report after conization and hysterectomy showed squamocellular carcinoma with microinvasive focuses. Interestingly, squamocellular carcinoma was found in the proximal uterine and adnexal structure, as well as intraepithelial and microinvasive lesions. *Conclusions*: Our study described a rare presentation of primary cervical SCC with unusual adnexal involvement. This pattern of tumor growth should be especially considered for patients who are proposed for sparing surgical procedures. A detailed and multidisciplinary approach for every patient is very important because unpredictable cases are present. However, they are rare.

## 1. Introduction

Cervical cancer is one of the most common gynecological malignancies. It affects about 500,000 women worldwide [1]. The most frequent histology type is squamocellular carcinoma (SCC), representing approximately 70–80% of all cervical cancers [1,2,3,4].

Tumor growth in cervical tissue was mostly locoregional in the stroma as a destructive, infiltrative pattern. In some cases, a growth pattern can be superfitial with intraepithelial progression and not extand stromal involvement. SCC usually infiltrates the surrounding cervical stroma with lymphogenic metastases in locoregional and, sometimes, distant lymph nodes. Advanced diseases could be presented with parametrial infiltration and ureter obstruction with consequent hydronephrosis [4].

A very uncommon and rare pattern is the proximal spreading of SCC with endometrial colonization [1]. More rarely, it constitutes further proximal spreading to adnexa. Such unusual tumor presentation should be described more because that kind of tumor growth could be mistaken with primary ovarian/adnexal tumors [5]. Considering very rare cases, such as metastasis, therapy options, and prognosis for these patients are still uncertain [1,6].

In this study, we represent microinvasive SCC with secondary endometrial and adnexal involvement, whereas the most invasive cancer behavior was in tubal segments. That kind of tumor growth was very unexpected and was not described in the previous literature.

## 2. Materials and Methods

In this study, we presented a 62-years-old patient who underwent surgery because of a primary cervical carcinoma detected on previous clinical and histopathology examinations. The patient was treated in the Clinic for Gynecologic and Obstetrics, University Clinical Centre of Serbia for the last three years. The following clinical and histopathology features were recorded: tumor size, presence of lymphovascular invasion (LVI), corpus, adnexal involvement, and lymph nodal status.

After detailed histomorphological analysis, we found synchronous cancer involvement in the endometrium and adnexa.

## 3. Results

### 3.1. Clinical Features

The patient has been in menopause for 12 years. She had one vaginal delivery when she was 21. In the last three years, the patient has noticed more extensive bleeding every month in different intensities and frequencies. Hypothyreosis and psoriasis are comorbidities.

A cervical biopsy was conducted two years ago after clinical examination and abnormal smear (group IIIB). Upon microscopic analysis, we found remarkable epithelial dysplasia (high-grade squamous intraepithelial lesion—H-SIL).

Because of refusing the suggested diagnostics and therapeutic procedures in the next period, the patient was monitored. A couple months ago, upon colposcopic examination, mucosal lesions were found as granular surfaces with punctiform discoloration and a slightly deformed cervical shape. Recidivancs H-SIL was colposopically confirmed. A histopathology report, after conization, showed squamocellular carcinoma with microinvasive focuses, which were also present on the apical surgical resection line.

Two months ago, the patient underwent radical hysterectomy with bilateral lymphadenectomy, where cervical microinvasive cancer was confirmed. Interestingly, squamocellular carcinoma was also found in the proximal uterine and adnexal structure as intraepithelial and microinvasive lesions.

### 3.2. Imaging Characteristics

**Figure 1:**
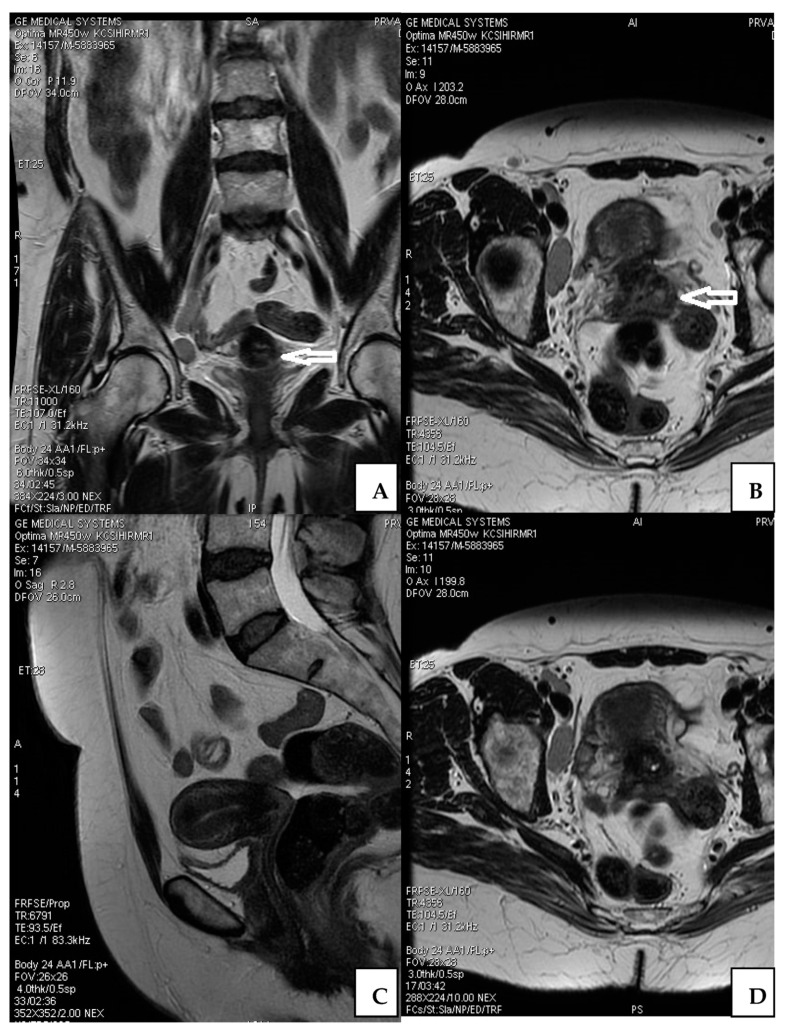
MRI of the abdomen and pelvis after conization. Cervical lesion (arrow) on diferent imaging presentations (**A**,**B**); Endometrium without detected tumor lesions (**C**); Adnexa without tumor lesion (**D**).

Abdomen and pelvis MRI examinations were conducted after conization, which showed a resting tumor in the cervical tissue (diameter 7 × 5 mm). The tumor spread to the proximal uterine segment without marked stromal infiltration. There was no tumor presence in parametrial tissue. The endometrium showed involutive characteristics. Many slightly enlarged lymph nodes were noticed, but without metastatic characteristics. Adnexal structures on imaging were not associated with suspected neoplastic lesions (Figure 1).

### 3.3. Squamocellular Carcinoma in the Cervix

The first histopathology analysis was conducted two years ago when we found a high squamous intraepithelial lesion (H-SIL) on the biopsy specimen. Citonuclear atypia was marked, with dominant epithelial stratification and remarkable mitosis in the upper epithelial layer. It was marked as cervical intraepithelial neoplasia (CIN) in gradus III, and it was also presented in the surrounding endocervical glands. After conization, beside H-SIL, we noticed microinvasive focuses of SCC in the upper parts of the cervical stroma. SCC with similar features was reported on specimen after hysterectomy.

**Figure 2:**
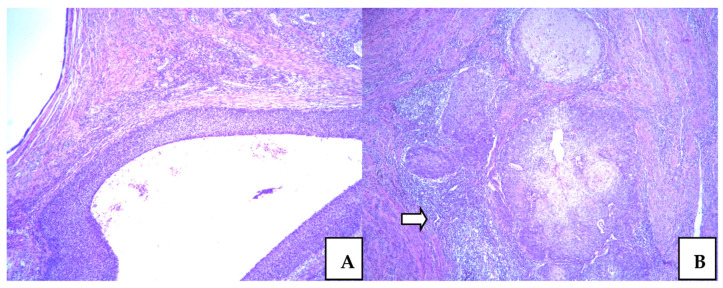
Squamocellular carcinoma in the cervix uteri. High grade epithelial displasia (H-SIL) surronding with tumor free endocervical tissue with inflammatory changes ((**A**). ×50); Squamocellular in situ cevical carcinoma with microinvasive foci (arrow) ((**B**). ×50).

Dysplastic high-grade epithelial lesions were predominant in all specimens. Microinvasive focus was in the subisthmical parts of cervical stroma measured 1mm deep. It was a very discrete focus presented as small cell clusters with atypical cancer cells, surrounded by intensive lymphocyte inflammatory reaction. The lymphovascular invasion was not found. These features are presented in Figure 2.

### 3.4. Squamocellular Carcinoma in the Endometrium

**Figure 3:**
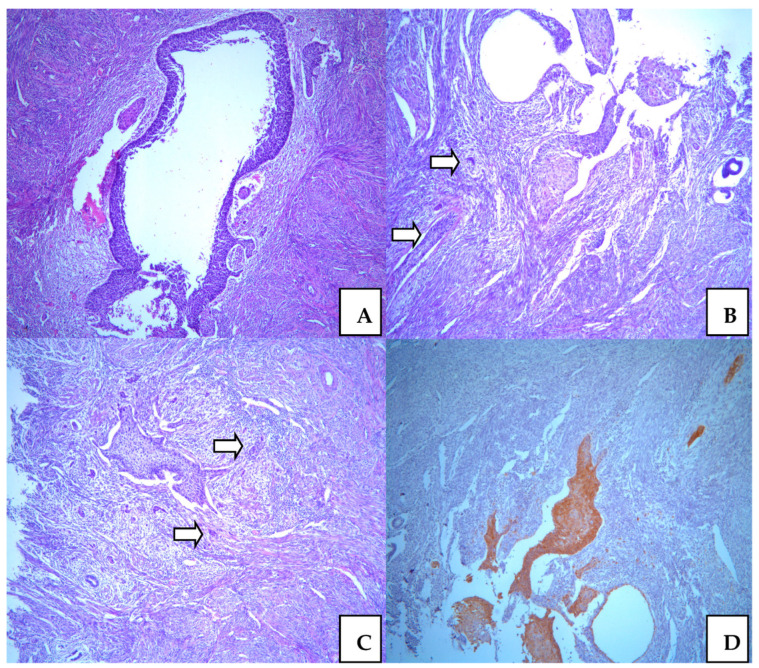
Squamocellular carcinoma in the uterine corpus with micoinvasive focus and surronding H-SIL colonization. Isthmic uterine part ((**A**). ×50); Endometrium with microinvasive foci (arrow) ((**B**). ×50, and ((**C**). ×50); Immunostaining with p16 antibody confirm cervical origin of microinvasive focuses in endometrium ((**D**). ×50).

The endometrium was associated with involutive features. In the myometrium, we noticed a couple of leiomyomas and adenomyomatosis without neoplastic lesions. Epithelial dysplasia was presented in the isthmic uterine part, with similar characteristics, such as a H-SIL lesion. The mostly whole upper endometrium was colonized with H-SIL, which showed remarkable atypia. In a few microscopic spots, we noticed microinvasive focuses in the myometrium, which are very similar to those in the cervical tissue. The depth of invasion was also less than 1mm. Differently from the cervix, there was lymphatic cancer invasion. Endometrial changes were the result of direct neoplastic extension from the cervix (Figure 3).

### 3.5. Squamocellular Carcinoma in the Adnexa

**Figure 4:**
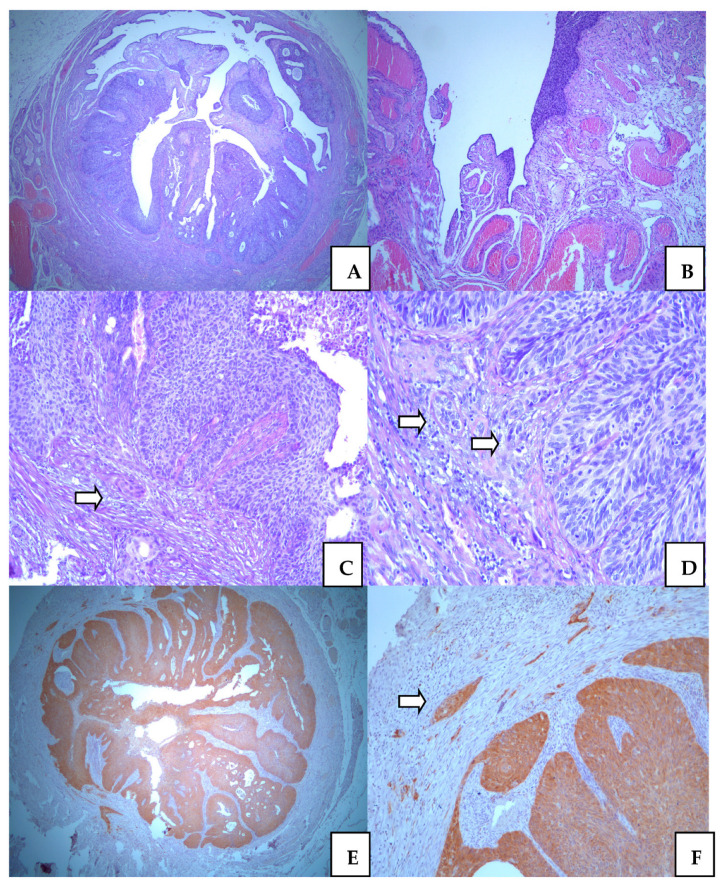
Squamocellular carcinoma in the uterine tuba. Intraepithelial neoplastic lesion replaces normal tubal epithelium ((**A**). ×25, and (**B**). ×50); Microinvasive foci (arrows) in surronding fibrous tissue of tubal wall ((**C**). ×100, and (**D**) ×100); Positive p16 immunostaining in neoplastic epithel with remarkable expression in micorinvasive foci (arrow) ((**E**). ×25, and (**F**). ×100).

Ovaries were without malignant features. There were benign, simplex cysts surrounded by slightly hyperthecosus ovarian stroma. In the right tubal epithelium, we found segments with remarkable intraepithelial cancer in in situ lesions. The altered epithelium was stratified, with marked atypia and increase mitotic figures. On the very extended series of histology sections, we noticed microinvasive SCC focuses in the tubal wall. The depth of invasion was not more than 1mm. However, unlike SCC, in the cervix, here we additionally observed invasion of lymphatic and vascular vessels. The microscopic pattern of SCC is more aggressive in tubal than in cervical tissue. Tubal involvement of SCC was presented in Figure 4. Additional extirpated lymph nodes did not show metastatic lesions.

## 4. Discussion

This case stands out because of its very interesting neoplastic growth pattern. Cervical cancer extraordinarily showed proximal spreading to the upper uterine segments. From our experience, endometrial colonization in the supraisthmic uterine segments was diagnosed in a few cases, but without adnexal involvement, and without such invasive behavior to the right of them.

Cervical cancers infrequently metastasize to the adnexa [7,8]. It has been established that the ovary is one of the most metastatic sites for many tumors [2,3,5]. There were not enough studies about the involvement of fallopian tubes, which could be the only metastatic site considering adnexa. A small number of studies report primary cervical SCC with secondary tubal capture [6]. These lesions were mostly intraepithelial and inconspicuous, measured less than 1mm, and were without invasive features [6]. The neoplastic epithelium was stratified with tall cells, hyperchromatic nuclei, and prominent mitoses that closely simulate serous tubal intraepithelial carcinoma (STIC) [6].

Endocervical adenocarcinoma is a more common histology type that secondarily involves adnexa, as opposed to SCC [2]. Adnexal presentation of primary endocervical invasive adenocarcinomas is typically unilateral and mostly cystic, with similar features as primary ovarian borderline tumours. [9]. The gastrointestinal histology subtype is the most frequent endocervical adneocarcinoma detected in adnexa, which can simulate metastatic features from digestive origin [2,9].

Many studies try to explain this rare metastatic behavior of cervical cancers. One hypothesis was based on genomic analysis, which reported simultaneous mutation in different uterine and adnexal parts. It could be confusing because it is difficult to determine primary tumor origin. A clonal relationship between synchronous tumor sites could be key for this tumor growth [6]. A study reported that these tumors are monoclonal, originating from the cervical mucosa with subsequent superficial spreading to the upper genital mucosa according to a loss of heterozygosity [10].

One theory was epithelial–mesenchymal transition (EMT), which allows tumor cells to increase their mobility and skip to distant structures where they continue their development. These cells become similar to cancer stem cells, lose their epithelial characteristics, and become more detached. After hosting in secondary sites, tumor cells are modified in the epithelial again and continue their proliferation as cancer cells [6,11]. Some authors reported the retrograde moving of tumor cells during retrograde menstruation. In that way, cancers cells reach adnexal structures and make new tumor focuses [6].

Ovarian stroma has structural characteristics suitable for metastasis homing. It secretes some factors, such as CXCR4, IL-8, and TNF-α, which can attract tumor cells to the ovarian area [6]. An expressed molecule—CD138—in cancer cells may participate in superficial spreading by regulating intercellular reactions. Cancer cells without CD138-expressed protein show infiltrative growth patterns [10].

Adnexal metastases from primary cervical carcinoma can appear after a long latent period. Such metastases are described over 11 years after initial diagnosis. It indicates the importance of careful follow-up for patients previously treated for cervical cancer [9,12].

## 5. Conclusions

Our study described a rare presentation of primary cervical SCC with unusual adnexal involvement. Previously reported cases mostly represent extensive invasive SCC cervical cancer with spreading in the proximal uterine parts. In this report, we emphasize the possibility of aggressive and unexpected tumor behavior in secondary sites, regardles to primary minor lesions. The superficial tumor spreading in the proximal uterine segment and the adnexa is not frequent. This pattern of tumor growth should be especially considered for patients who are proposed for sparing surgical procedures. A detailed and multidisciplinary approach for every patient is very important because unpredictable cases are present. However, they rare are.

## Data Availability

Data is contained within the article.
